# Hyperthermia treatment of tumors by mesenchymal stem cell-delivered superparamagnetic iron oxide nanoparticles

**DOI:** 10.2147/IJN.S94255

**Published:** 2016-05-09

**Authors:** Tammy L Kalber, Katherine L Ordidge, Paul Southern, Michael R Loebinger, Panagiotis G Kyrtatos, Quentin A Pankhurst, Mark F Lythgoe, Sam M Janes

**Affiliations:** 1 Lungs for Living Research Centre, UCL Respiratory, University College London, UK; 2 UCL Centre for Advanced Biomedical Imaging, Division of Medicine, University College London, UK; 3 Healthcare Biomagnetics Laboratory, University College London, London, UK

**Keywords:** mesenchymal stem cells, SPIONs, hyperthermia, MRI, tumor therapy

## Abstract

Magnetic hyperthermia – a potential cancer treatment in which superparamagnetic iron oxide nanoparticles (SPIONs) are made to resonantly respond to an alternating magnetic field (AMF) and thereby produce heat – is of significant current interest. We have previously shown that mesenchymal stem cells (MSCs) can be labeled with SPIONs with no effect on cell proliferation or survival and that within an hour of systemic administration, they migrate to and integrate into tumors in vivo. Here, we report on some longer term (up to 3 weeks) post-integration characteristics of magnetically labeled human MSCs in an immunocompromized mouse model. We initially assessed how the size and coating of SPIONs dictated the loading capacity and cellular heating of MSCs. Ferucarbotran^®^ was the best of those tested, having the best like-for-like heating capability and being the only one to retain that capability after cell internalization. A mouse model was created by subcutaneous flank injection of a combination of 0.5 million Ferucarbotran-loaded MSCs and 1.0 million OVCAR-3 ovarian tumor cells. After 2 weeks, the tumors reached ~100 µL in volume and then entered a rapid growth phase over the third week to reach ~300 µL. In the control mice that received no AMF treatment, magnetic resonance imaging (MRI) data showed that the labeled MSCs were both incorporated into and retained within the tumors over the entire 3-week period. In the AMF-treated mice, heat increases of ~4°C were observed during the first application, after which MRI indicated a loss of negative contrast, suggesting that the MSCs had died and been cleared from the tumor. This post-AMF removal of cells was confirmed by histological examination and also by a reduced level of subsequent magnetic heating effect. Despite this evidence for an AMF-elicited response in the SPION-loaded MSCs, and in contrast to previous reports on tumor remission in immunocompetent mouse models, in this case, no significant differences were measured regarding the overall tumor size or growth characteristics. We discuss the implications of these results on the clinical delivery of hyperthermia therapy to tumors and on the possibility that a preferred therapeutic route may involve AMF as an adjuvant to an autologous immune response.

## Introduction

Hyperthermia >43°C is capable of inducing cell death both directly, by inducing apoptosis, and indirectly, by protein denaturation or DNA damage.^1^ Hyperthermia has also been shown to adversely affect the fluidity and stability of cellular membranes, the function of transmembrane transport proteins, and cell surface receptor expression.^1^ Notably, tumor cells are more sensitive to sudden increases in temperature than normal cells, making hyperthermia an attractive therapeutic tool.^2^

Hyperthermia in superparamagnetic iron oxide nanoparticle (SPION)-laden tumors can be induced by the presence of a rapidly alternating magnetic field (AMF).^3^ SPIONs are nanosized particles with a diameter typically ranging between 50 and 100 nm. They consist of one or more crystalline cores of superparamagnetic iron (Fe^3+^) oxide, which are typically 5–10 nm in diameter, surrounded by a biocompatible coating such as starch, dextran, carboxydextran, or citric acid. SPION cores are small enough that the net magnetocrystalline anisotropy energy, which is proportional to the volume of the particle, is comparable to the environmental thermal bath energy at, or below, room temperature. This leads to thermally induced reversals of the net magnetization, which is the phenomenon of superparamagnetism. The production of heat can be generated through magnetic hysteretic and relaxation losses (Néel relaxation) as well as through physical rotation (Brownian relaxation) under the influence of an AMF.^4^,^5^

SPION-induced hyperthermia within tumors has been achieved previously but has relied on direct injection of milligram concentrations of iron into superficial tumors,^6^–^11^ which limits the approach to only the accessible superficial tumors, such as melanomas. Although functionalization of SPIONs with antibodies,^12^–^15^ antibody fragments,^16^,^17^ or peptides^18^–^21^ has been used to enhance the accumulation of SPIONs in tumors, there have been problems with pharmacokinetics, immunogenicity, and toxicity.

We, and others, have shown that mesenchymal stem cells (MSCs) migrate to and incorporate into tumors.^22^–^26^ We therefore propose the possibility of using MSCs as cell carriers to deliver SPIONs to multiple, difficult-to-reach sites, as in the case of a metastatic malignant disease. However, this presupposes that the heating character of SPIONs within the MSCs is retained after internalization, as well as over a prolonged period of time that would be needed for cell targeting prior to hyperthermia treatment. Retention of the heating potential may also be considered to be a necessary safety element for any potential therapeutic interventions based on magnetic field hyperthermia. For instance, it may well be difficult to prove, a priori, that an introduced MSC might not, on arrival at a tumor site, itself differentiate into a tumor cell – thereby potentially exacerbating rather than improving the situation. Although our previous data suggest that normal MSCs do not affect tumor growth in an orthotopic lung tumor model,^27^ in such a case, the ability to kill by hyperthermia, the introduced MSC might well be seen to be an imperative fail-safe characteristic.

MSCs have been shown to actively take up and retain SPIONs from a loaded cell culture medium.^22^ Although there have been reports in the literature of dose-dependent effects on the health of MSCs,^28^,^29^ our group and others have shown that it is possible to achieve SPION labeling at a level that does not affect the survival, proliferation, or differentiation potential of MSCs.^22^,^30^ Furthermore, we have demonstrated that MSCs maintain their migratory capacity to pulmonary metastases after SPION labeling, with subsequent infiltration of the tumor.^22^ We have also shown that SPION-loaded MSCs can be targeted to specific areas via magnetic targeting.^31^–^33^ We have therefore hypothesized that the tumor-targeting potential of MSCs could be exploited to deliver SPIONs to tumors for subsequent hyperthermia therapy. However, we have not previously established whether SPIONs within the MSCs degrade or otherwise lose their heating potential during the period between their introduction into the body and their subsequent arrival at the tumor. The present study is designed to address this question by exploring the time-dependent heating characteristics of SPION-loaded MSCs in a long-term, viable, animal model.

The present study defines the contribution of size and chemical coating of six different SPIONs on MSC uptake and AMF-induced hyperthermia. We assess the role of cellular internalization of each SPION on the subsequent level of AMF-induced hyperthermia and choose the SPION with the most suitable characteristics. We then assess the therapeutic possibility of using SPION-loaded MSCs as a delivery vector for hyperthermia therapy in an in vivo subcutaneous tumor model in immunosuppressed nude mice. The immunosuppressed model is chosen for two reasons: 1) to assess the physical effect of heating on cell death and tumor volume rather than combine (and possibly confuse) the outcomes with downstream mediators such as immune responses and 2) to enable accurate measurements of both tumor size, via external calipers, and heat generation, via skin thermocamera imaging.

## Materials and methods

### Superparamagnetic iron oxide nanoparticles

FluidMAG-CT (polymer matrix: citric acid), 50 nm and 100 nm; FluidMAG-CMX (carboxymethyldextran), 50 nm; FluidMAG-DX (dextran), 50 nm; and FluidMAG-D (starch), 50 nm, nanoparticles were obtained from Chemicell (Berlin, Germany). Ferucarbotran^®^ (carboxydextran) 60 nm-sized particles were obtained from Meito Sangyo Co., Ltd. (Nagoya, Japan).

The hydrodynamic diameter and ζ-potential of each iron oxide were determined by using a Nanosizer ZS90 (Malvern Instruments, Malvern, UK). A Quantum Design superconducting quantum interference device (SQUID) VSM magnetometer (Quantum Design Inc., San Diego, CA, USA) was used to assess the magnetic properties of the nanoparticles.

One milliliter solutions of 1 mg/mL were made up in water for both FluidMAG and Ferucarbotran to assess particle-heating capacities.

### Tissue culture

Human MSCs were purchased from the Tulane Centre for Gene Therapy, New Orleans, LA, USA, and were cultured in alpha minimum essential media (α-MEM) supplemented with 16% heat-inactivated fetal bovine serum (FBS), L-glutamine (4 mM), penicillin G (50 U/mL), and streptomycin (50 µg/mL), all of which were purchased from Thermo Fisher Scientific (Waltham, MA, USA).

Human OVCAR-3 ovarian carcinoma cancer cells were obtained from the Metabolic and Molecular Imaging Group, MRC Clinical Sciences Centre, Imperial College, London (originally from the ATCC^®^ HTB-161™). Cells were cultured in RPMI 1640 media (Thermo Fisher Scientific) supplemented with 10% FBS, L-glutamine (4 mM), penicillin G (50 U/mL), and streptomycin (50 µg/mL).

All cells were grown in T175 flasks (Thermo Fisher Scientific) in a humidified incubator at 37°C with 95% air and 5% CO_2_. The cell lines used within this study have been purchased from known cell providers, and are an expansion of the original primary cell culture. These cell lines are therefore not considered relevant material under the Human Tissue Act and do not require ethics approval.

### SPION labeling, phenotyping, visualization, and iron quantification of MSCs

MSCs were incubated overnight (12 hours) with 0.5 mg/mL of SPION solution in 20 mL normal α-MEM cell culture media. Following this, they were washed multiple times with 10 mL phosphate-buffered saline (PBS), until excess SPIONs had been removed. Adipogenic and osteogenic differentiation of MSCs was performed as previously described.^34^,^35^ Prussian blue staining of fixed cells (4% paraformaldehyde) was performed using freshly prepared 2% potassium ferrocyanide and 2% hydrochloric acid (Sigma-Aldrich, Co., St Louis, MO, USA) and incubated for 10 minutes. Images were taken using a Zeiss Axiovert S100 microscope (Carl Zeiss Meditec AG, Jena, Germany) with QCapture software (QImaging, Surrey, BC, Canada).

Cells for iron quantification or in vivo injection were trypsinized and counted, and an aliquot of 5×10^5^ cells were pelleted for in vitro heating and magnetometry analysis.^22^ Room temperature SQUID magnetization analysis was used to measure the amount of iron oxide. The samples were saturated in a field of 7 T and the moment was measured. The saturated moment was then compared with a sample of known iron oxide mass in order to quantify the iron oxide cell loading mass. The remainder was resuspended in PBS at a concentration of 1×10^6^ cells/mL to be stained with CellTracker™ CM-DiI (Thermo Fisher Scientific) for in vivo injection. A total of 5 µM DiI (5 µL) was added and left for 15 minutes on ice prior to washing.

### Animals and tumor model

All animal studies were approved by the University College London Biological Services Ethical Review Committee and licensed under the UK Home Office regulations and the Guidance for the Operation of Animals (Scientific Procedures) Act 1986 (Home Office, London, UK). Animal weights and tumor monitoring were recorded every 2 days. Magnetic resonance imaging (MRI) scanning was carried out with animal monitoring equipment. All procedures were carried out under breathing anesthesia, and all mice were sacrificed by schedule 1; 1×10^6^ OVCAR-3 cells were mixed with 5×10^5^ SPION-labeled MSCs in 100 µL serum-free RPMI media and injected subcutaneously into the flanks of 6–8-week-old female immunosuppressed Balb/c nu/nu mice (n=4, two not heated and two heated – MSCs labeled with Ferucarbotran). Once palpable, tumors were measured in three orthogonal dimensions using calipers. Tumor volumes were estimated assuming an ellipsoid shape using the formula:

Volume=length×width×depth×π/6.

### Magnetic resonance imaging

All images were acquired on a 9.4 T horizontal bore Agilent NMR system (Agilent, Palo Alto, CA, USA) using a 39 mm radio frequency coil (RAPID Biomedical GmbH, Wurzberg, Germany). On days 14, 18, and 21, mice were anesthetized with an isoflurane/oxygen mixture using a warm air blower to maintain the body core temperature at 37°C. A fast spin-echo multislice sequence using the following parameters was used to evaluate the presence of SPIONs within the tumor; repetition time =1,500 ms, echo time =10 ms, field of view =40×40 mm^2^, averages: one, matrix size: 128×128, 12 contiguous 2 mm thick transverse slices covering the whole lower abdomen were used to ensure complete tumor coverage.

### Magnetic alternating current hyperthermia device

The magnetic alternating current hyperthermia (MACH) system (Resonant Circuits Limited, London, UK) generated an AMF of 10 mT at 1.05 MHz in both a three-turn copper solenoid coil (3.5 cm diameter for mice) and a six-turn copper solenoid coil (2.7 cm diameter for Eppendorf phantoms). Cold water was run through the center of the copper to prevent ohmic heating of the coil, so that this did not transfer any environmental heat to any object placed inside the coil.

SPION Eppendorf phantom preparations were typically heated for 20 minutes from room temperature to assess the heating capacity of the particles at set concentrations. Thermal plateaus were reached in this time. MSCs labeled with SPIONs in ~100 µL total volume (cells plus residual fluid) were also heated from room temperature until they reached a plateau temperature.

On days 15, 17, 19, and 20, mice were anesthetized with an isofluorane/oxygen mixture and received either 20 minutes of hyperthermia therapy or 20 minutes of sham therapy with the AMF switched off. Treatment was performed in a thermostat-regulated warming cabinet (MediHeat, Peco Services, Cumbria, UK), with the inside temperature maintained at 37°C, to keep the core mouse temperature stable.

A Luxtron m3300 fiber-optic thermometry system was used to measure the temperature changes in vitro and the core and surface tumor temperature changes in vivo (Luxtron Co, Santa Clara, CA, USA). TrueTemp™ software (Luxtron Co) was used to acquire temperature data over each time course. For the final in vivo treatment session, an infrared thermal imaging camera (InfraTec VarioCAM^®^ hr research 780) with IRBIS 3 software (InfraTec, Dresden, Germany) was also used to monitor the temperature of the tumor to compare the fiber-optic data recordings. The core temperature was then used to correct for any heating that may have been caused by alterations in body temperature due to the warming box.

### Histology

After the final MRI, the mice were sacrificed and tumors removed and fixed in 4% formalin. Tumors were processed and embedded in paraffin wax blocks and sectioned in 3 µm slices. Adjacent slices from each tumor were dewaxed and rehydrated with water and stained with hematoxylin and eosin (H&E) (Sigma-Aldrich Co.) and Perl’s Prussian blue for iron or counterstained with 4′,6-diamidino-2-phenylindole (DAPI; Sigma-Aldrich Co.) for HSP70 immunohistochemistry and DiI immunofluorescence.

H&E sections were incubated with hematoxylin solution for 1 minute and then in freshly prepared eosin Y for 30 seconds. Perl’s sections were incubated in freshly prepared 2% potassium ferrocyanide and 2% hydrochloric acid, as for fixed cells, for 10 minutes and then counterstained with 0.1% nuclear fast red (Sigma-Aldrich Co.) for 5 minutes. HSP70 immunohistochemical slides were submitted to antigen retrieval by two, 5-minute cycles in boiling 10 mM sodium citrate in a microwave oven. The endogenous peroxidase activity was blocked with 3% H_2_O_2_ in PBS. For the detection of HSP70, the slides were incubated for 1 hour with monoclonal antibody sc-1060 (Santa Cruz Biotechnology Inc., Dallas, TX, USA) at a 1:100 dilution at room temperature. Biotinylated secondary antibody and avidin–biotin complex and 3,39-diaminobenzidine (Vectastain ABC Kit – Vector Laboratories, Burlingame, CA) were applied for visualization of the immunoreaction. Slides were counterstained with hematoxylin. Omission of the primary antibody was considered as a negative control. Histopathological alterations were assessed on H&E-stained sections. Sections were then dehydrated with alcohol and passed through histoclear (Thermo Fisher Scientific) and mounted with DPX (VWR, Poole, UK).

DiI sections were incubated with 300 nM DAPI and mounted using Vectashield mounting media (Vector, Peterborough, UK). Images were obtained using an Olympus BX40 microscope (Olympus Corporation, Tokyo, Japan) with QCapture software. Composite images of whole tumors were made from multiple single 100× images using Adobe Photoshop CS (Adobe Systems Incorporated, San Jose, CA, USA). Random slices throughout the tumors were then used to obtain the percentage area of both Perl’s and DiI staining using ImageJ software.^36^

## Results

### MSC endocytosis of SPION is dependent on particle coating

**Figure 1:**
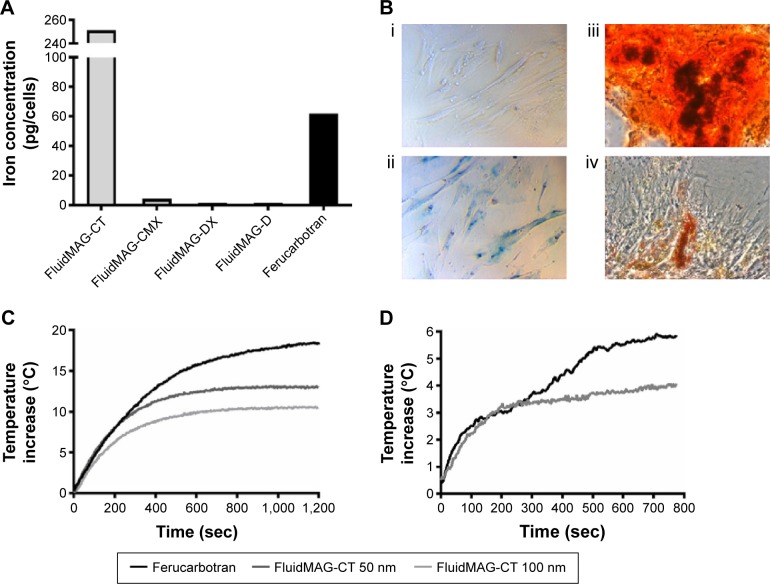
SPION uptake in MSCs and their corresponding magnetic heating characteristics. **Notes:** (**A**) SQUID measurements of the concentration of iron oxide per mesenchymal stem cell (MSC) after overnight incubation of 0.5 mg/mL of FluidMAG-CT (citric acid – 50 nm), FluidMAG-CMX (carboxylmethyldextran), FluidMAG-DX (dextran), FluidMAG-D (starch), and Ferucarbotran (carboxydextran). (**B**) MSCs take up SPIONs without affecting their phenotype; (i) MSCs in culture; (ii) Perl’s Prussian blue staining of MSCs after overnight culture with Ferucarbotran nanoparticles; (iii) differentiation to osteoblasts, Alizarin Red S staining; (iv) differentiation to adipocytes, Oil Red O staining. (**C**) Representative fiber-optic thermometry measurements of Ferucarbotran and FluidMAG-CT (50 nm- and 100 nm-sized SPIO particles) at 1 mg/mL. (**D**) Representative fiber-optic thermometry measurements of cell heating of a 5×10^5^ MSC pellet after overnight incubation of 0.5 mg/mL of FluidMAG-CT 50 nm or Ferucarbotran (representative of three experiments). **Abbreviations:** SQUID, superconducting quantum interference device; SPION, superparamagnetic iron oxide nanoparticle; sec, seconds.

Our first aim was to evaluate the uptake of the six different SPIONs within MSCs. The chosen SPIONs had a range of synthetic coatings with and without functional groups. FluidMAG SPIONs were supplied with a range of particle coatings, citric acid (CT), caroboxymethyldextran (CMX), dextran (DX), and starch (D). We compared cellular uptake of the different coated FluidMAG particles and evaluated them against the carboxydextran-coated Ferucarbotran. SQUID measurements demonstrated FluidMAG-CT to have the highest uptake in cells with a maximal uptake of 250 pg/cell (Figure 1A). The hydrodynamic diameter of FluidMAG-CT (50 nm) was 75 nm (slightly larger than the 50 nm specified) and the ζ-potential was −52 mV, which reflects the negative charge of the functional carboxyl groups (COOH) on the surface. When a carboxyl group is deprotonated, a carboxylate anion is formed giving the particle an overall negative charge, and it is this charge that aids the uptake of the particle across the cell wall.^37^,^38^ The hydrodynamic diameter of Ferucarbotran particles was 77 nm and the ζ-potential was −23 mV. Ferucarbotran also has carboxyl groups present,^39^ but to a lesser degree, and this particle showed an intermediate uptake of ~60 pg/cell (Figure 1A). Particles that were either neutral in charge or did not have reactive functional groups (such as dextran- and starch-coated particles) showed little uptake into cells (Figure 1A). Uptake of iron within the MSCs does not affect their differentiation potential in vitro (Figure 1B).

### AMF-induced SPION heating is dependent on particle size

FluidMAG-CT particles were obtained in two particle sizes, 50 and 100 nm, and the influence of particle size on heating was evaluated. Representative heating changes from baseline room temperature for 1 mg/mL Ferucarbotran (60 nm) and FluidMAG-CT, 50 and 100 nm-sized particles, are shown in Figure 1C. Ferucarbotran exhibited the highest heating capacity with a maximal increase in temperature of 18.5°C from baseline temperature (Figure 1C). The FluidMAG-CT 50 nm particle exhibited a temperature increase from the baseline of 13.1°C, while the FluidMAG-CT 100 nm particle showed the lowest temperature rise at 10.6°C (Figure 1C). This demonstrates that particle size significantly affects AMF-induced heating for FluidMAG-CT. The 100 nm FluidMAG-CT particle was not tested further.

The heating curves were further analyzed with respect to the intrinsic loss parameters (ILPs) of the particles.^40^ Following recommended analytical methods,^41^ the data yielded estimates ILPs of 2.4±0.1 nH m^2^ kg^−1^ for Ferucarbotran and 2.1±0.1 nH m^2^ kg^−1^ for the FluidMAG-CT 50 nm particle.

### SPION retention of AMF-induced heating properties after MSC endocytosis

The heating capabilities of both the FluidMAG-CT (50 nm) and Ferucarbotran SPIONs were tested using a pellet of 5×10^5^ SPION-labeled MSCs. Despite the fourfold increase in SPION concentration per cell in FluidMAG-CT-labeled cells (Figure 1A), Ferucarbotran still exhibited a greater rise in temperature by 5.9°C compared to 4.1°C (Figure 1D). It is notable that the concentration of Ferucarbotran in the cell pellet (60 pg/cell for 5×10^5^ cells in a volume of 100 µL) corresponded to ~300 µg/mL and that the observed temperature rise was ~30% of the value achieved in the 1 mg/mL Ferucarbotran phantom. This suggests that the heating mechanism is the same for Ferucarbotran SPIONs internalized within the MSCs, as it is in the water-based fluid.

The superior heating characteristics of Ferucarbotran are further exemplified by contrast with the performance of the FluidMAG-CT. The concentration of the latter was higher at ~1.2 mg/mL (250 pg/cell for 5×10^5^ cells in a volume of 100 µL), but the cells showed a markedly reduced heating curve compared to that of the 1 mg/mL phantom, suggesting breakdown or degradation of the particles upon cell internalization. Ferucarbotran was therefore determined to be the superior heating particle for this application and was taken into the in vivo studies.

A final aspect is that the heating curves observed in the Ferucarbotran-loaded MSC pellets exhibited an unexpected two-step characteristic (Figure 1D). Despite careful scrutiny, we were not able to unambiguously determine the origin of this behavior, and it remains unexplained. One possibility is that it was related to a thermally induced change in the evaporation behavior of the sample.

### AMF application to tumors containing SPION-loaded MSCs shows macroscopic heating and cell necrosis

**Figure 2:**
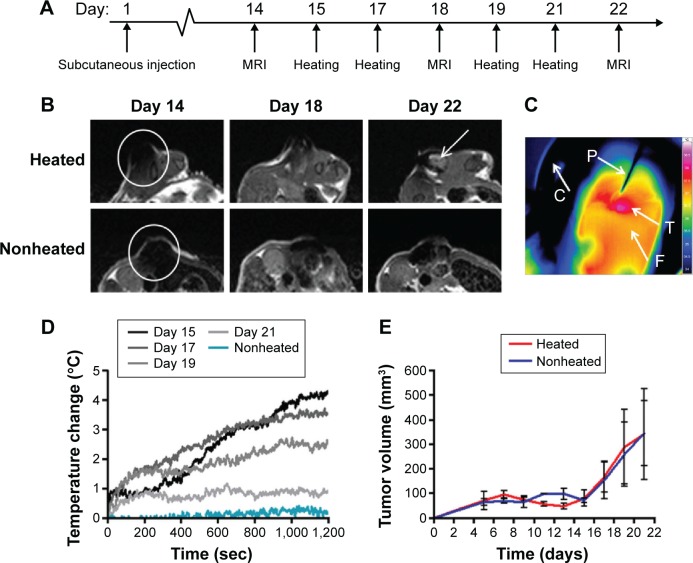
Murine model of OVCAR-3 tumor growth, with and without magnetic heating. **Notes:** (**A**) Experimental plan of MRI and AMF heating. (**B**) T2 weighted MR images of a heated and nonheated OVCAR-3 tumor coinjected with Ferucarbotran-labeled MSCs, at days 14 (prior to heating), 18, and 22 (postheating) (white arrow indicates signal recovery). (**C**) Thermocamera image of a heated tumor (T) on the flank of a nude mouse (F) with C indicating the MACH coil and P the fiber-optic thermometry probe. (**D**) Fiber-optic thermometry measurements of a heated tumor over days 15, 17, 19, and 21 and a representative nonheated tumor and core body temperature. (**E**) Post-innoculation OVCAR-3 tumor growth for both heated and nonheated tumors. **Abbreviations:** MRI, magnetic resonance imaging; AMF, alternating magnetic field; MR, magnetic resonance; MACH, magnetic alternating current hyperthermia; sec, seconds; MSCs, mesenchymal stem cells.

Subcutaneous tumors were initiated by coinjecting 1 million OVCAR-3 cancer cells with 0.5 million MSCs containing 60 pg/cell Ferucarbotran SPIONs. At day 14, the tumors measured ~100 mm^3^ (~100 µL). Thereafter, they entered a rapid growth phase, growing up to 300 mm^3^ (~300 µL) by day 22. Our previous study demonstrates that while there is expansion of the tumor cell population, the MSC population shows no detectable proliferation but maintains viability.^22^ After 14 days of growth, tumors were assessed by MRI and then subjected to two rounds of AMF heating (days 15 and 17). Tumors were reimaged on day 18, followed by two additional rounds of AMF heating (days 19 and 21), and a final MRI image (day 22) was taken before sacrifice (Figure 2A).

MRI of the OVCAR-3 tumors performed on day 14 (before any AMF heating) showed complete signal loss throughout the whole tumor, indicating no loss of SPIONs from the cells, despite being within the tumor for 2 weeks (Figure 2B). This hypointensity artifact in the MRI data is an effect of superparamagnetism of the particles, which are magnetically saturated within the scanner, leading to a locally perturbed magnetic field around the particle and a subsequent loss of measurable signal. At the day 15 of AMF hyperthermia treatment, fiber-optic thermometry readings and thermoimaging indicated an AMF-induced heat increase of 4.3°C after application for 20 minutes – a surface temperature of 41.5°C (Figure 2C and D). On repeat heating on days 17 and 19, the fiber-optic thermometry values gradually fell, until on the fourth and final heating (day 21), and the temperature after 20 minutes of AMF application was close to that of the nonheated control (Figure 2D).

MRI performed at day 22 demonstrated signal recovery in the heated OVCAR-3 tumors, compared to the nonheated tumors. This signal recovery likely indicates a loss of SPIONs within these tumors from MSC death and subsequent iron removal from the tumor (Figure 2B). No significant differences in tumor size or growth were noted for the heated tumors compared to the nonheated controls throughout the study (Figure 2E).

### Hyperthermia treatment results in reduced content of both MSCs and SPIONs within tumors

**Figure 3:**
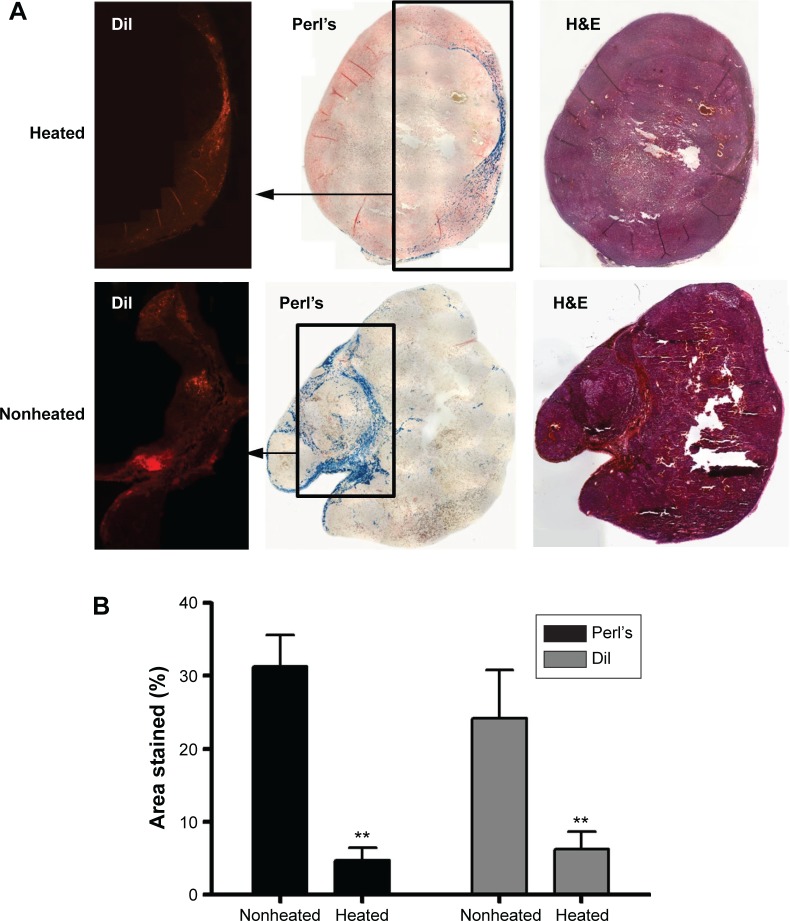
Immunohistochemistry data from a human OVCAR-3 tumor model, with and without magnetic heating. **Notes:** (**A**) Sections from a representative tumor stained with DiI cell tracker dye, Perl’s Prussian blue iron stain, and H&E on day 22, derived from a coinjection, on day 1, of Ferucarbotran-labeled MSCs and OVCAR-3 tumor cells that were subsequently either heated or nonheated. (**B**) Percentage stained areas of representative slices throughout both heated and nonheated tumors for both Perl’s and DiI staining (error bars are SEM and **P<0.01). **Abbreviations:** H&E, hematoxylin and eosin; MSCs, mesenchymal stem cells; SEM, standard error of the mean.

While hyperthermia treatment did not affect tumor growth, the signal recovery seen on MRI at day 22 in the heated tumors suggested the loss of iron oxide. We confirmed histologically that OVCAR-3 tumors receiving AMF hyperthermia therapy had significantly less Perl’s Prussian blue staining (*P*<0.01) and significantly reduced DiI staining compared to that in the nonheated tumors (*P*<0.01). This indicates the loss of both iron and MSCs from the tumors (Figure 3A and B), consistent with MSC death due to heating and subsequent clearance from the tumor tissue. In the nonheated tumors, Perl’s staining suggested that MSCs were not evenly distributed throughout the tumor and that instead the MSCs tended to be situated in viable tumor areas, namely, near the periphery.

A corollary of the observation of reduced Perl’s and DiI staining in the heated tumors, and of our interpretation of this as being the result of AMF-induced MSC death and subsequent clearance, is the implication that the tumor environment is one that allows active macrophage infiltration and trafficking. If this is the case, then it may be argued that macrophage clearance would apply also to any dead cells within the tumor – in which case, the observation of sustained Perl’s and DiI staining in the nonheated tumor tissues may be taken as indirect evidence that in the absence of AMF treatment, the SPION-loaded MSCs remain viable and active within the tissue.

HSP70 staining showed no differences from controls, which may be due to the transient expression of HSP70 that would have been at its highest directly after the first and second AMF treatment 4 days prior to tumor removal.^42^ However, HSP70 staining could also have been confounded by the locality of the iron particles to any peroxidase staining. However, subsequent staining with a Texas Red fluorescent secondary antibody (sc-3919, Santa Cruz Biotechnology) also did not show staining above that of controls (data not shown).

## Discussion

We have shown the potential of MSC delivery of SPIONs combined with AMF in delivering hyperthermia treatment to tumors. Although hyperthermia produced by SPIONs after direct injection has shown potential for tumor ablation, this technique is only deliverable to superficial tumors and is unlikely to supersede the therapies that are in effect at the moment. However, MSCs have the potential to deliver SPIONs to tumors deep within the body at metastatic sites.

In our studies, we have demonstrated that the size and chemical coating of the SPIONs influence human MSC uptake in a similar manner to that we had previously seen for rabbit MSCs.^31^ The greater uptake of FluidMAG-CT is most likely due to the increased number of carboxyl groups and the subsequent greater charge on the particle surface.^37^,^38^ However, despite its relatively low concentration after cell internalization (60 pg/cell compared to 250 pg/cell), it was Ferucarbotran, originally an MRI liver contrast agent,^39^ that exhibited the best heating characteristics. This is a reassuring result, given that Ferucarbotran has recently been used in a number of other hyperthermia studies.^40^,^43^,^44^

The observed in vitro temperature rise of 5.9°C from baseline using Ferucarbotran in a cell pellet was not quite matched in the tumor model, where the surface temperature rise was more moderate at 4.3°C – albeit the internal temperature may have been higher. No significant hot spots were shown on the thermocamera, which showed a near-uniform temperature increase across the whole tumor, although histology suggested that the SPIO-loaded MSCs were not heterogeneously spread throughout the tumor tissue.

Although somewhat beyond the intended scope of the present study, it is interesting to examine the physiological outcomes of AMF treatments on the mice. Despite the 4.3°C rise in surface temperature, the hyperthermia treatment did not result in measurable retardation of tumor growth. A possible explanation for this is that the overall MSC and SPION concentration was simply not enough to cause large-scale tumor cell death. A contributing factor here could have been the increase in tumor volume causing an already inhomogeneous distribution of MSC to be further dispersed, thereby reducing the local concentration of SPIONs. Another possibility is that the initial heating was effective enough to induce destruction of the MSC population and loss of the SPIONs, but not widespread enough to retard the proliferation of OVCAR-3 cells during the fast-growth phase. In any case, it was difficult to assess any local increase in necrosis that may have been caused, given the inherently high baseline necrosis of the tumor model and the small number of animals used.

Nevertheless, both MRI at day 22 and subsequent histology sections showed reduced iron and DiI staining in the AMF samples, suggesting heating-induced MSC death with subsequent clearance of both SPIONs and MSCs, compared to that in the nonheated tumors. At the same time, there were still some single MSCs remaining after four heating treatments. Histology showed that these remaining MSCs were surrounded by cancer cells. Given the previously observed inhomogeneity in the distribution of MSCs in the nonheated tumors, this might be an indication that cell death and removal were predominantly affected in the MSC clusters, rather than in isolated MSCs. If this were the case, it would appear that to promote intracellular heating, and therefore improve prospects of killing adjacent cancer cells, it may be important to find ways to promote the clustering or aggregation of magnetically labeled cells at the target tumor site.

MRI was found to be a good way of evaluating MSC death. Dead MSCs and exogenous SPIONs are cleared by tumor macrophages leading to changes in SPION concentration and distribution within tumors, with areas of MRI signal recovery correlating well with areas of low Perl’s Prussian blue and DiI staining by histology in hyperthermia-treated tumors. However, the retention of the MRI signal loss in the nonheated tumors suggests that we can label MSCs with iron for at least 2 weeks, that they are viable (otherwise, they would have been cleared), and that they are retained within the tumor tissue over this time. The fact that we also show tumor heating of up to 4.3°C at day 14 indicates that Ferucarbotran retains its heating properties over this period and is not degraded within the cells.

Although we have not replicated the near or complete regression of tumors shown in studies using direct injection of SPIONs,^6^–^8^ the effects we have seen were with concentrations that were several-fold less. Furthermore, the absence of any immune response in our mouse model may be significant. A recent study by Basel et al^45^ evaluated mouse monocyte/macrophage-like cells as iron carriers that migrate toward disseminated peritoneal pancreatic cancer cells after intraperitoneal injection. They showed an increase in survival time in the AMF-treated mice compared to controls, using a combination of cell labeling and AMF characteristics that resulted in an overall tumor heating of 4°C – similar to that observed in this study. Basel et al explained their result partly on the basis of recruitment of various immune cells to the tumors.^46^–^49^ Hyperthermia causes the expression of HSPs that in turn induces antitumor immunity, activating antigen-presenting cells to migrate into and kill both heated and nonheated tumors, including metastatic disease.^50^–^53^ However, in this study, histology staining for HSP70 was inconclusive, which may be due to the fact that the highest temperature increase occurred at the first AMF treatment. As HSP70 expression is transient, this would decrease over the subsequent AMF treatments and may have fully recovered prior to tissue removal.^42^ We therefore surmise that it is possible that we are heating to temperatures capable of triggering an immune response but that, by using human cancer cells in an immunodeficient mouse, we are observing only the pure effect of heating and therefore do not gain from the immunogenic effect.

It therefore appears to be the case that the immune system has a much larger role to play in tumor cell death than from direct hyperthermia than previously thought. Nonetheless, our human MSCs are more efficient iron carriers than the mouse monocyte-/macrophage-like cells used in the study by Basel et al, as they showed no iron breakdown and retained efficient heating qualities for >2 weeks after injection, allowing for a long time window for AMF treatments in patients; however, phagocytic cells metabolize iron within days. Therefore, further studies using mouse MSCs in an immunocompetent mouse model would greatly benefit this AMF treatment approach.

Looking ahead, it is interesting to speculate that AMF treatments may find a role in the treatment of metastatic cancers that is not based on thermoablation and cellular necrosis, but rather on moderate levels of hyperthermia, leading to individual-cell apoptosis and instigation of the body’s own immune response. Recent results from our own laboratory show the ability of hyperthermia to elicit cell-by-cell controlled apoptosis,^54^ and studies by Ito et al^51^,^52^ and Sato et al^55^ suggest that even local hyperthermia could induce a form of vaccination that may be effective against metastatic disease, negating the need for the distribution of MSCs throughout whole tumor tissues. As such, we may, in the future, be looking to magnetic field hyperthermia as an adjuvant, rather than an intervention in its own right.

## Conclusion

In summary, although we have by necessity been restricted to a limited number of small animal experiments, we have nevertheless demonstrated in vivo that MSCs can be used as carriers of Ferucarbotran SPIONs, that these labeled MSCs can remain viable and can be retained in the tumor tissue over a matter of weeks, and, that in that time, the internalized Ferucarbotran can remain intact and preserve its heating capacity within the cell.
